# Assessing the Effects of Childhelp’s Speak Up be Safe Child Abuse Prevention Curriculum for High School Students

**DOI:** 10.1007/s40653-021-00353-1

**Published:** 2021-05-03

**Authors:** Marisol Juarez Diaz, Diane Moreland, Wendy Wolfersteig

**Affiliations:** Southwest Interdisciplinary Research Center (SIRC), Watts College of Public Service and Community Solutions, School of Social Work, Arizona State University, Phoenix, AZ 85004 USA

**Keywords:** Adolescent, Child abuse and neglect, Child maltreatment, Evidence-based practice, Prevention, Youth

## Abstract

**Purpose:**

This study examined the Childhelp Speak Up Be Safe (CHSUBS) child abuse prevention curriculum for high school students and addressed a gap in evidence-based child maltreatment prevention programs. CHSUBS is grounded in theory and was developed to 1) provide students with the skills they need to prevent or interrupt child abuse, bullying, and neglect, and 2) increase student knowledge about safety related to abuse.

**Methods:**

Utilizing a cluster-randomized controlled trial design, the three high schools were randomly assigned to participate in the CHSUBS curriculum or the control group. Survey items measured the efficacy of the curriculum in grades 9 through 12. Surveys were implemented at baseline, immediately after the intervention, and after 6 months for a follow-up. Analyses included exploratory factor analyses and a paired samples *t–*test to determine whether increases in child maltreatment knowledge and resistance skills were gained.

**Results:**

Findings showed positive significant results that child maltreatment knowledge and resistance skills were significantly different from pre to post for the CHSUBS group and showed no significant control group changes.

**Conclusions:**

High school students in the CHSUBS group appeared to gain both child maltreatment knowledge and resistance skills. Future studies on prevention programming for high school students might show results that lead to a reduction in child maltreatment and an increase in better health outcomes for adolescents.

## Introduction

Child maltreatment (CM; i.e., emotional abuse, physical abuse, sexual abuse, and neglect) is a widespread social and public health epidemic (Sedlak et al., 2010), and while preventable, continues to be highly damaging to children’s well-being over the life course (Black et al., 2017). Globally, it is estimated that up to 1 billion children aged 2 to 17 years have experienced physical, sexual, or emotional violence in the past year (Hillis et al., 2016). In the United States, new and concerning federal child abuse and neglect data show an increase in the number of victims who suffered CM for the first time since 2015, in addition to an increase in the number of child fatalities due to CM in 2018 (U.S. Department of Health and Human Services [DHHS], 2020).

Age is a critical characteristic and risk factor related to CM, and adolescence is an especially risky period in that it holds the possibility of new and compounding adverse experiences associated with negative outcomes (Felitti et al., 1998). In 2017, the national rate of victimization for U.S. high school students was 9.1 per 1000 youth, equating to more than 90,000 youth who experience CM during their high school years (DHHS, 2019). Furthermore, a national survey captured lifetime CM rates among U.S. adolescents (14–17 years old), with 38.1% reporting lifetime maltreatment, 23.9% experiencing emotional abuse, 18.4% experiencing neglect, and 18.1% reporting physical abuse (Finkelhor et al., 2015).

Adolescents are distinct as they are nearing the end of their childhood and can give a more comprehensive estimate of child maltreatment’s total prevalence. In addition, adolescents are extra vulnerable to poly-victimization (the impact of cumulative forms of victimization), which is common and destructive to adolescent functioning and development. One study documented that nearly 80% of the participants reported at least one lifetime victimization, and adolescents aged 15–18 experienced *15 or more* victimization types (Finkelhor et al., 2009). A different study reported that 70% of adolescents aged 14–17 endorsed a history of any witnessed violence and 17-year-olds reported that most sexual assaults (69%) occurred as an adolescent, whereas relatively few (7%) occurred in early childhood, age 0–5 (Saunders & Adams, 2014). Although overall data trends indicate that rates of physical and sexual abuse have declined over the past 20 years (Finkelhor, 2020), that is not the case with neglect, a form of maltreatment understudied in adolescents (Vanderminden et al., 2019), with 1 in 7 youth reporting neglect at some point in their lives, a substantial increase from 49% in 1990 to 75% in 2017 (Child Trends, 2019).

In 2018, around 7.8 million children were referred to Child Protective Services because of alleged CM (DHHS, 2020). These CM rates are dangerous given the damaging consequences on children’s development and the social and economic impact. Longstanding evidence confirms the relationship between CM and increased risks for major causes of morbidity and mortality across the life course and generations (Felitti et al., 1998). These increased dangers include criminal behavior, substance use, poor mental health, suicidality, sexual risk behaviors, and teen pregnancy (Abajobir et al., 2018; Hahm et al., 2010; Herrenkohl et al., 2013, Proctor et al., 2017; Yun et al., 2010). In addition to promoting risky adolescent behaviors, CM undermines youth socio-emotional development (Alink et al., 2012) and self-esteem (Oshri et al., 2017). Therefore, the experience of multiple types of CM reflects an overall relentlessness that places youth at an elevated risk for problem behaviors and socio-emotional instability.

A recent study found that risky behavior and consequences such as teen pregnancy and substance use occur significantly more often among adolescents who experience an investigation of CM, regardless of whether the report is substantiated (Kugler et al., 2019). Beyond these amplified individual-level risks, child maltreatment presents an economic burden on the government and healthcare systems. Peterson et al. (2018) estimate the cost of CM in the U.S. to be approximately $428 billion annually. These economic and social effects stress the importance of prevention strategies that encourage safe, stable and nurturing relationships with trusted adults to help children be resilient.

### School-Based Prevention Programming

School-based child safety programs are an increasingly common method to address CM (Finkelhor et al., 2018). Schools provide daily contact with children and parents, and studies show that children who participate in school-based programs are more likely to report instances of sexual abuse than children who did not participate in a prevention program (Walsh et al., 2018). Research has found that youth have made substantial gains in abuse-related knowledge and skills due to participating in child abuse prevention programming (Dale et al., 2016; Finkelhor et al., 2018). Educating high school students about this topic is essential to prevent CM from occurring in the future and reduce the impact of CM that might have already occurred. As barriers persist for reporting, the need for making child protection and police involvement more child-friendly and providing education and guidance from educators and health professionals is essential.

Childhelp’s Speak Up Be Safe (CHSUBS) prevention curriculum is designed for pre-kindergarten through grade 12 and focuses on disrupting all forms of child abuse and maltreatment, including neglect and cyberbullying (Diaz et al., 2021). Research supports the effectiveness of child safety and prevention efforts that focus on the broad spectrum of victimizations youth suffer (Finklehor et al., 2009). With developmentally appropriate materials, the CHSUBS curriculum teaches age-appropriate definitions and universal strategies to prevent poly-victimization and foster self-protective skills. It was developed to 1) provide students with the skills they need to prevent or interrupt child abuse, bullying, and neglect, and 2) increase student knowledge about safety as related to abuse. Moreover, grounded in theory, for example, ecological systems (Bronfenbrenner, 1979) and resiliency (Masten, 2014; Zimmerman, 2013), each CHSUBS lesson (at each grade level) includes activities to provide practice on the application of skills across multiple contexts for students to distinguish what they need to stay safe and healthy. For example, for grades 9–12, the lessons include teaching knowledge, safety rules, and resistance strategies through scenarios, discussions, and worksheets that go home to the parents in English and Spanish describing what their child learned in the lessons. Along with completing the CHSUBS K-8 study (under review), the results from the CHSUBS high school cluster-randomized controlled trial advances opportunities for building a primary prevention approach to reach all students at any grade level.

## Method

The CHSUBS high school cluster-randomized controlled trial (RCT) occurred from a 6-year collaborative partnership between a non profit (Childhelp) and a research center (Center). Childhelp, who owns the curricula, partnered with the Center to test the CHSUBS curriculum’s efficacy by strategically collaborating with the high school staff (teachers, counselors, administrators) in a school district in the Southwest to help with the RCT. The purpose of this RCT was to examine the efficacy of the CHSUBS high school curriculum using a pre-, post-, and 6-month follow-up design.

### Schools and Randomization

Three Southwest schools were invited to participate and were randomly assigned to either the implementation or control group conditions. For the three high schools, the two with smaller enrollment numbers were combined as one high school group; then, each of the two high school groups was randomly assigned to either a control or intervention group using a coin toss. Because the school with the largest number of students was randomly selected first and assigned to the implementation group condition, the remaining two schools were next assigned to the control group.

#### *Sample*

Sample participants were selected from a high school district that covers two growing cities in the Southwest. The three public high schools had similar student demographics, including approximately the same number of females and males, and the average age in both groups was 15/16 years. Participants included students in grades 9 through 12. Students in the schools assigned to the implementation condition received the curriculum and completed three surveys (i.e., pre-, post-, 6-month follow-up). Survey administration was conducted online and facilitated by high school teachers using Qualtrics software. Students who attended the two schools assigned to the control condition did not receive the program but completed the three surveys online. Both implementation and control schools were offered the curriculum free of charge for the next school year. There were a total of 887 participants (implementation = 416; control = 471) with matched pre- and post-surveys used in this analysis. Due to COVID-19 school closures in March and the move to online learning, there was a low response rate in May of returned follow-up surveys (*N* = 307) with even fewer (*N* < 100) matched across all three times. Thus, data analyses were conducted for matched pre- and post-survey participants. Demographic data, which included gender, grade, age, and ethnicity, were collected (see Table 1).

**Table 1:** Demographics

**Variables**	**Implementation**		**Control**	
	*N*		*N*	
**Sample Size**	416		471	
	*M (SD)*		*M (SD)*	
**Age**	15 (1.31)		16 (1.23)	
	*N*	%	*N*	%
**Gender**				
Male	192	46.2	197	41.8
Female	192	46.2	216	45.9
Option to Self-Describe	9	2.2	7	1.5
Prefer Not to Answer	9	2.2	13	2.8
**Race/Ethnicity**				
Hispanic/Latino	170	40.9	179	38
White	196	47.1	241	51.2
African American or Black	53	12.7	46	9.8
American Indian or Alaskan Native	27	6.5	27	5.7
Asian or Pacific Islander	15	3.6	15	3.2
Other Ethnicity	23	5.5	17	3.6
Prefer Not to Answer	25	6	13	2.8

*Note* Gender does not add up to 100.0% due to "Prefer Not to Answer" or missing responses. Race/Ethnicity does not add to 100.0% due to students being able to select more than one category

#### *Procedures*

##### Curriculum Development

In 2014–2015, Childhelp initiated a partnership with the Center to complete an evidence-informed update to the curriculum and instruments, a process that included a curriculum pilot test in 2016 (Diaz et al., 2021). In the CHSUBS curriculum, students learn how to be safe through various concepts, key terms, and safety rules depending on their grade level. Students in high school learn six-resistance strategies using the acronym RESIST: Run, Escape, Scream, Ignore, Stay Away, and Tell. The curriculum was developed for two 45-min sessions at each grade level to be facilitated by teachers or social workers with groups of approximately 25 to 30 students. The CHSUBS curriculum is delivered through presentation and discussion materials available through an online platform. Content and delivery are based on several research areas, including child development, learning styles, social theory, and child maltreatment prevention.

### Measures

The pre-, post-, and follow-up survey items were based on the learning objectives and key terms taught within the CHSUBS curriculum. Measures were designed and initially tested in 2016–2017 during a pilot study of the curriculum with 282 students in grades 9–12. A readability analysis was conducted for each item, using the Flesch-Kinkaid Grade Level Test, to ensure age appropriateness, and the surveys were revised before the cluster-randomized controlled trial.

The survey items were the same for all grades (9–12). Items included demographic, child maltreatment, and resistance strategy items. The same items were included on the pre-, post-, and follow-up surveys, with additional program satisfaction-related items, included only on the post- and follow-up questionnaires for students in the implementation group. There were 16-child maltreatment knowledge items included in the surveys ranked on a 6-point Likert scale ranging from 1 (*strongly disagree*, to 5 (*strongly agree*). The surveys also included six questions about the RESIST strategies: Run, Escape, Scream, Ignore, Stay Away, and Tell. The students were given the first letter of each RESIST item and asked to identify the correct strategy from the presented answer choices.

**Table 2:** Results from a Factor Analysis of Child Maltreatment Knowledge Scale Items

Item	Factor Loading
There are rules that can help keep me safe.	.437
Being intentionally mean over the Internet is a form of bullying.	.601
Neglect is a type of abuse.	.638
Many states have anti-bullying laws that include cyberbullying.	.394
There are safe adults.	.407
Abusers are very good at hiding their bad intentions from the people whom they abuse.	.374
Child abuse means to hurt a child on purpose.	.510
Neglect happens when children are not given what they need to be healthy and safe.	.640
Once I send a message using a digital device, I have no control over how it gets shared.	.498
Cyberbullies never tease or harass people online. (R)	.445
Physical abuse often leaves a bruise, a broken bone, a cut, or a burn.	.387
Sexual abuse can include talking about sexual activity.	.516
Bullying, emotional abuse, and sexual abuse can all happen over the Internet.	.601
Emotional abuse is also called verbal or psychological abuse.	.710
If an adult shows his or her private body parts to a teenager this is abusive behavior.	.550
Keeping yourself safe involves understanding various types of abuse.	.689
Eigen Value	4.594
% of Total Variance	28.714

*Note. N* = 847. Reverse-scored item is denoted with an (R).

#### *Procedure and Implementation Protocol*

All protocols and instruments for data collection were reviewed and approved by the Social Behavioral Institutional Review Board (IRB) at Arizona State University. Opt-out forms were sent home to parents, letting them know that their child would participate in a research study and gave information on how to opt their child out of the surveys. Before starting the cluster-randomized controlled trial, teachers completed an online curriculum facilitator training, which included universal modules on child abuse and neglect and modules specific to the grade levels they were facilitating. Teachers also participated in three 1.5-hour trainings about tracking opt-out forms and facilitating online access to pre-, post-, and follow-up surveys.

#### *Data Collection*

Pre-surveys were administered in the fall of 2019. Due to additional teacher training, pre-survey administration for the implementation group was delayed and therefore caused a shorter than planned amount of time between pre- and post-surveys. The implementation (CHSUBS) group received the post-survey 3-weeks after the pre-survey. CHSUBS group students completed the pre-survey before receiving the first lesson and the post-survey after the second lesson. For the control group, there were 7-weeks between pre- and post-survey administration. In the late Spring of 2020, during the COVID-19 pandemic when students were remote learning, the follow-up survey was administered to the control group approximately 5-months later and the implementation group approximately six-months later. All three surveys were administered electronically using a Qualtrics link provided by teachers on the day of each survey. Students were asked to enter their unique student identification numbers at the beginning of each online survey. Qualtrics electronic data files were downloaded and imported into SPSS for cleaning and analysis. During data cleaning, the unique student identification number was used to match each students pre-, post-, and follow-up surveys.

#### *Data Analysis*

A factor analysis using principal component analysis was conducted on the 16-child maltreatment knowledge items. Observation of the scree plot suggested one factor with all 16-child maltreatment knowledge items (see Table 2). The analysis yielded one child maltreatment knowledge scale that explained 28.714% of the variance. Internal consistency was examined for the child maltreatment knowledge scale using Cronbach’s alpha. The alpha was acceptable at .82 (Tavakol & Dennick, 2011). Next, each student’s cumulative child maltreatment knowledge score was calculated across the 16 items for each survey. Paired sample *t*-tests were conducted within each group to determine if significant child maltreatment knowledge changes occurred from the pre- to post-survey.

In addition to examining child maltreatment knowledge changes, resistance strategy gains were investigated. Responses to the resistance strategy items were dichotomized to reflect correct (1) and incorrect (0) responses. An initial factor analysis using principal component analysis was conducted on the six-resistance strategy items. After examining the scree plot, the analysis suggested one factor with all six-resistance strategy items (see Table 3). The six items were Run, Escape, Scream, Ignore, Stay Away and Tell. The analysis yielded a RESIST scale and explained 45.833% of the variance. Internal consistency was examined for the resistance scale using Cronbach’s alpha. The alpha was acceptable at .73 (Tavakol & Dennick, 2011). Next, a cumulative resistance strategy score was calculated for each student across the six items. A mean correct score out of 100% was generated for each student for pre- and post-survey responses. Pre- to post-survey overall mean resistance strategy scores were examined using a paired sample *t*-test to determine if significant resistance strategy score changes occurred after the post-survey within each group.

**Table 3:** Results from a Factor Analysis of RESIST Scale Items

Item	Factor Loading
R =	0.588
E =	0.820
S =	0.728
I =	0.575
S =	0.779
T =	0.514
Eigen Value 2.750	
% of Total Variance 45.833	

*Note. N* = 830

## Results

### Child Maltreatment Knowledge

Paired sample *t-*tests were conducted to examine differences between pre- and post-survey scores. For the CHSUBS group, there was a significant difference in CM knowledge scores between the pre-survey (*M* = 3.91, *SD* = .46) and post-survey (*M* = 4.00, *SD* = .61); *t*(392) = −3.47, *p* = .001, *d* = .18. The CHSUBS group post-survey CM knowledge score was significantly higher than the pre-survey score. For the control group, there was not a significant difference in CM knowledge scores between the pre-survey (*M* = 4.02, *SD* = .48) and post-survey (*M* = 4.02, *SD* = .59); *t(*439) = −.20, *p* = .845, *d* = 0.01. (see Table 4).

**Table 4:** Results for Paired-Sample t-Tests of Overall Child Maltreatment Knowledge Scale Scores

		*n*	*M*	*SD*	*t*	*df*	*p*	Cohen’s *d*
Implementation								
Pre-score		393	3.91	.46				0.18
Post-score			4.00	.61				
Pre-Post score					−3.47	392	.001	
Control								
Pre-score		440	4.02	.48				0.01
Post-score			4.02	.59				
Pre-Post score					−0.20	439	.845	

### Resistance Strategies

For the implementation group, there was a significant difference in RESIST scale scores between the pre-survey (*M* = .85, *SD* = .23) and post-survey (*M* = .95, *SD* = .18); *t*(364) = −8.04, *p* = .000, *d* = 0.42; the implementation post-survey RESIST scale score was significantly higher than the pre-survey score. For the control group, there was not a significant difference in RESIST scale scores between the pre-survey (*M* = .84, *SD* = .23) and post-survey (*M* = .86, *SD* = .23); *t*(398) = −1.28, *p* = .203, *d* = .06 (see Table 5).

**Table 5:** Results for Paired-Samples t-Tests of Overall RESIST Scale Scores

		*n*	*M*	*SD*	*t*	*df*	*p*	Cohen’s *d*
Implementation								
Pre-score		365	.85	.23				0.42
Post-score			.95	.18				
Pre-Post score					−8.04	364	.000	
Control								
Pre-score		399	.84	.23				0.06
Post-score			.86	.23				
Pre-Post score					−1.28	398	.203	

### Program Satisfaction Ratings

**Figure 1:**
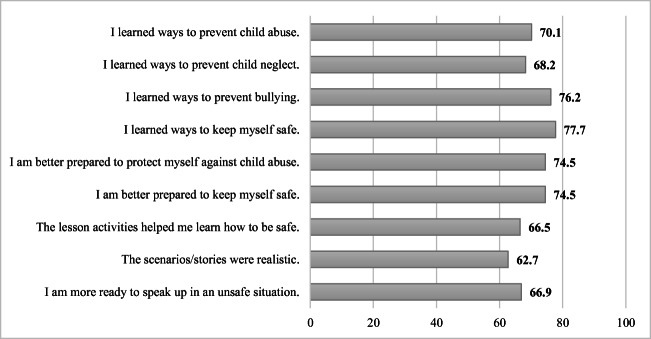
Implementation of Students’ Program Satisfaction Ratings for “Agree” and “Strongly Agree.”

Overall, the implementation students who received the CHSUBS program reported positive satisfaction ratings that ranged from 62.7% to 77.7% for those who responded they *agree* or *strongly agree* with items on how satisfied they were with the lessons (see Figure 1). Many students reported **learning new ways** to prevent abuse, neglect, bullying, and **keeping myself safe**. Three quarters of students’ reported being **better prepared** after the program to protect and keep themselves safe. Lastly, two thirds of CHSUBS students reported that they are **more ready to speak up in an unsafe situation** after participating in the CHSUBS curriculum (66.9% responded *agree* or *strongly agree*). However, there is room for improvement as 62.7% of students indicated that the scenarios and stories used in the surveys could have been more realistic and relatable. A suggestion was to have the scenarios include discussions of college hazing and bullying, and empowering students to use safety strategies that can be utilized in various life stages.

## Discussion

### Value of This Evidence

The evidence of efficacy for the CHSUBS curriculum for grades 9-12 was established with this cluster-randomized controlled trial design and the resulting significant differences between the implementation pre and post-survey scores and the lack of change for the control group from pre- to post results. Because there are few child maltreatment programs geared for universal groups of high school students, this adds to the literature and the list of evidence-based programs that now exist for those purposes. These results help fill that prevention programming gap of options available for high school aged youth to learn more about child abuse and neglect what many forms it takes (e.g., sexual abuse, bullying) and the resistance strategies to deal with CM situations.

The CHSUBS students showed significant positive changes in both knowledge gains and resistance strategies for dealing with CM situations. This skill-building approach provides teenagers with an active role in their protection and personal safety, based on their new knowledge and resistance strategies. That the CHSUBS students also reported positive levels of satisfaction with the lessons adds to this program being a suitable means to deliver the tough messages of CM in all its forms.

Unfortunately, many existing evidence-based CM prevention programs are focused on single harm and created for younger audiences. Adding the more broadly based CHSUBS CM prevention program to the list of evidence-based choices should help to invigorate schools, districts, and community-based organizations into choosing to present CM information to teenaged youth, knowing this is a developmentally appropriate and researched means of delivery. Students are empowered to have a role in their safety by learning critical skills to respond appropriately to all types of abuse, including neglect. These CHSUBS results also demonstrate a universal program applicable to students of all racial/ethnic backgrounds, with approximately half of the study participants identifying as non-white.

Furthermore, school districts that encompass grades kindergarten to 12 now have the CHSUBS program available as one comprehensive, evidence-based curriculum for use at all levels rather than meld different programs for different-aged audiences. As a program that uses various learning options, CHSUBS has a fully manualized curriculum available online. These high school results showed that CHSUBS imparts knowledge and skills that help students in grades 9-12 to deal with child maltreatment issues.

### Limitations

Despite the strengths of the current findings, this study is not without limitations. The current study employed a rigorous, scientific design with a 6-month follow-up survey to test the duration of program effectiveness. However, due to barriers resulting from COVID-19 school closures and remote learning, the small number of returned follow-up surveys was less than ideal for analysis, and one of the two control schools dropped out due to COVID complications. Thus, although the follow-up was completed, results are not reported, and the researchers suggest additional evaluations to demonstrate that learning is maintained over time.

Also, as mentioned in the data collection summary, the amount of time between pre- and post-surveys for the implementation group was less than ideal. Navigating school scheduling needs (e.g., school events, mandatory standardized testing, holiday breaks) and adding teacher training ensuring fidelity with well-trained facilitators created a delay in the pre-survey administration. It, therefore, caused a shorter timeframe between pre- and post-survey than planned. This shorter period might favor showing an increase in knowledge, suggesting that more evaluations should be conducted.

Furthermore, results relied on the self-reported data from youth taught by different high school teachers. While the CHSUBS curriculum has extensive lesson plans for standardized implementation with well-trained facilitators, the researchers could not observe all the high school lessons being taught and concede there is always variation in how individual facilitators deliver the content, pointing to a possible future challenge in replicating results.

## Conclusions

CM is a serious public health issue, and a particular need exists for CM prevention programs to target high school youth given that current programs primarily focus on elementary children, are single-harm focused, and are not evidence-based. This study’s findings indicated that participation in CHSUBS increased high school students’ identification of key safety-related resistance strategies. These findings contribute to the literature on the importance and implications of teaching adolescent high school youth how to interrupt and prevent abuse to increase personal safety. Evidence-based programs are becoming a standard requirement for funders and drive policy formation and future program development, and prevention programs like CHSUBS have the promise to prevent a sizable portion of CM cost-effectively. Future studies on prevention programming for high school students might show results that lead to a reduction in CM, an increase in better health outcomes for youth, and prompt advocates to address the gap in prevention programming for high school youth.

## References

[CR1] Abajobir AA, Kisely S, Williams G, Strathearn L, Najman JM (2018). Risky sexual behaviors and pregnancy outcomes in young adulthood following substantiated childhood maltreatment: Findings from a prospective birth cohort study. The Journal of Sex Research..

[CR2] Alink LRA, Cicchetti D, Kim J, Rogosch FA (2012). Longitudinal associations among child maltreatment, social functioning, and cortisol regulation. Developmental Psychology.

[CR3] Author, M. J., Wolfersteig, W., Moreland, D., Yoder, G., Dustman, P., & Harthun, M. L. (2021). Teaching youth to Resist Abuse: Evaluation of a Strengths-Based Child Maltreatment Curriculum for High School Students. *Journal of Child & Adolescent Trauma*. 10.1007/s40653-020-00304-2, Teaching Youth to Resist Abuse: Evaluation of a Strengths-Based Child Maltreatment Curriculum for High School Students. 10.1007/s40653-020-00304-2 PMC7900375 33692874

[CR4] Black MM, Walker SP, Fernald LCH, Andersen CT, DiGirolamo AM, Lu C, McCoy DC, Fink G, Shawar YR, Shiffman J, Devercelli AE, Wodon QT, Vargas-Barón E, Grantham-McGregor S, Lancet Early Childhood Development Series Steering Committee (2017). Early childhood development coming of age: Science through the life course. Lancet (London, England).

[CR5] Bronfenbrenner, U. (1979). The ecology of human development. Cambridge: Harvard University Press.

[CR6] Child Trends. (2019). *Child maltreatment*. https://www.childtrends.org/indicators/child-maltreatment.

[CR7] Dale, R., Shanley, D. C., Zimmer-Gembeck, M. J., Lines, K., Pickering, K., White, C. (2016). Empowering and protecting children by enhancing knowledge, skills and well-being: A randomized trial of Learn to BE SAFE with Emmy™. *Child Abuse & Neglect 51*, 368–378. 10.1016/j.chiabu.2015.07.016 26360708

[CR8] Diaz, M. J., Wolfersteig, W., Moreland, D., Yoder, G., Dustman, P., & Harthun, M. L. (2021). Teaching youth to Resist Abuse: Evaluation of a Strengths-Based Child Maltreatment Curriculum for High School Students. *Journal of Child & Adolescent Trauma,**14*(1), 141–149. 10.1007/s40653-020-00304-2. 10.1007/s40653-020-00304-2 PMC7900375 33692874

[CR9] Felitti VJ, Anda RF, Nordenberg D, Williamson DF, Spitz AM, Edwards V, Koss MP, Marks JS (1998). Relationship of childhood abuse and household dysfunction to many of the leading causes of death in adults: The adverse childhood experiences (ACE) study. American Journal of Preventive Medicine.

[CR10] Finkelhor D, Ormrod RK, Turner HA (2009). Lifetime assessment of poly-victimization in a national sample of children and youth. Child Abuse & Neglect.

[CR11] Finkelhor D, Turner HA, Shattuck A, Hamby SL (2015). Prevalence of childhood exposure to violence, crime, and abuse: Results from the National Survey of Children's exposure to violence. JAMA Pediatrics.

[CR12] Finkelhor, D., Saito, K., & Jones, L. (2018). *Updated trends in child maltreatment*. 2016 Crimes against children research center. University of New Hampshire. Retrieved from http://www.unh.edu/ccrc/pdf/Updated%20trends%202016.pdf

[CR13] Finkelhor D (2020). Trends in adverse childhood experiences (ACEs) in the United States. Child Abuse & Neglect.

[CR14] Hahm HC, Lee Y, Ozonoff A, Van Wert MJ (2010). The impact of multiple types of child maltreatment on subsequent risk behaviors among women during the transition from adolescence to young adulthood. Journal of Youth and Adolescence.

[CR15] Herrenkohl TI, Hong S, Klika JB, Herrenkohl RC, Russo MJ (2013). Developmental impacts of child abuse and neglect related to adult mental health, substance use, and physical health. Journal of Family Violence.

[CR16] Hillis, S., Mercy, J., Amobi, A., & Kress, H. (2016). Global prevalence of past-year violence against Children: A Systematic Review and Minimum Estimates. *Pediatrics*. 10.1542/peds.2015-4079, Global Prevalence of Past-year Violence Against Children: A Systematic Review and Minimum Estimates. 10.1542/peds.2015-4079 PMC6496958 26810785

[CR17] Kugler KC, Guastaferro K, Shenk CE, Beal SJ, Zadzora KM, Noll JG (2019). The effect of substantiated and unsubstantiated investigations of child maltreatment and subsequent adolescent health. Child Abuse & Neglect.

[CR18] Masten, A.S. (2014) Global Perspectives on Resilience in Children and Youth. *Child Development 85*(1):6–20. 10.1111/cdev.12205 24341286

[CR19] Oshri A, Carlson MW, Kwon JA, Zeichner A, Wickrama KKAS (2017). Developmental growth trajectories of self-esteem in adolescence: Associations with child neglect and drug use and abuse in young adulthood. Journal of Youth and Adolescence.

[CR20] Peterson C, Florence C, Klevens J (2018). The economic burden of child maltreatment in the United States, 2015. Child Abuse and Neglect..

[CR21] Proctor LJ, Lewis T, Roesch S, Thompson R, Litrownik AJ, English D, Arria AM, Isbell P, Dubowitz H (2017). Child maltreatment and age of alcohol and marijuana initiation in high-risk youth. Addictive Behaviors.

[CR22] Saunders BE, Adams ZW (2014). Epidemiology of traumatic experiences in childhood. Child and Adolescent Psychiatric Clinics of North America.

[CR23] Sedlak AJ, Mettenburg J, Basena M, Petta I, McPherson K, Greene A, Li S (2010). Fourth National Incidence Study of child abuse and neglect (NIS–4): Report to congress.

[CR24] Tavakol M, Dennick R (2011). Making sense of Cronbach's alpha. International Journal of Medical Education.

[CR25] U.S. Department of Health & Human Services, Administration for Children and Families, Administration on Children, Youth and Families, & Children's Bureau. (2020). *Child Maltreatment*, 2018 https://www.acf.hhs.gov/cb/report/child-maltreatment-2018.

[CR26] U.S. Department of Health & Human Services, Administration for Children and Families, Administration on Children, & Youth and Families, Children's Bureau. (2019). *Child Maltreatment*, 2017 https://www.acf.hhs.gov/cb/report/child-maltreatment-2019.

[CR27] Vanderminden J, Hamby S, David-Ferdon C, Kacha-Ochana A, Merrick M, Simon TR, Finkelhor D, Turner H (2019). Rates of neglect in a national sample: Child and family characteristics and psychological impact. Child Abuse & Neglect.

[CR28] Walsh K, Zwi K, Woolfenden S, Shlonsky A (2018). School-based education programs for the prevention of child sexual abuse: A Cochrane systematic review and meta-analysis. Research on Social Work Practice.

[CR29] Yun I, Ball J, Lim H (2010). Disentangling the relationship between child maltreatment and violent delinquency: Using a nationally representative sample. Journal of Interpersonal Violence.

[CR30] Zimmerman, M.A. (2013). Resiliency theory: A strengths-based approach to research and practice for adolescent health. *Health Education & Behavior, 40*(4), 381–383. 10.1177/1090198113493782 PMC3966565 23863911

